# Parkinson-Related LRRK2 Mutation R1628P Enables Cdk5 Phosphorylation of LRRK2 and Upregulates Its Kinase Activity

**DOI:** 10.1371/journal.pone.0149739

**Published:** 2016-03-01

**Authors:** Yang Shu, Jie Ming, Pei Zhang, Qingzhi Wang, Fengjuan Jiao, Bo Tian

**Affiliations:** 1 Department of Neurobiology, Tongji Medical School, Huazhong University of Science and Technology, 13 Hangkong Road, Wuhan, Hubei Province, 430030, P. R. China; 2 Central laboratory, Affiliated Hospital of Jiangsu University, 438 Jiefang Road, Zhenjiang, Jiangsu Province, 212000, P. R. China; 3 Wuhan Union hospital, Tongji Medical School, Huazhong University of Science and Technology, 1277 Jiefang Avenue, Wuhan, Hubei Province, 430022, P. R. China; 4 Institute for Brain Research, Collaborative Innovation Center for Brain Science, Huazhong University of Science and Technology, 13 Hangkong Road, Wuhan, Hubei Province, 430030, P. R. China; 5 Key Laboratory of Neurological Diseases, Ministry of Education, 13 Hangkong Road, Wuhan, Hubei Province, 430030, P. R. China; Van Andel Institute, UNITED STATES

## Abstract

**Background:**

Recent studies have linked certain single nucleotide polymorphisms in the *leucine-rich repeat kinase 2 (LRRK2)* gene with Parkinson’s disease (PD). Among the mutations, *LRRK2* c.4883G>C (R1628P) variant was identified to have a significant association with the risk of PD in ethnic Han-Chinese populations. But the molecular pathological mechanisms of R1628P mutation in PD is still unknown.

**Principle Findings:**

Unlike other LRRK2 mutants in the Roc-COR-Kinase domain, the R1628P mutation didn’t alter the LRRK2 kinase activity and promote neuronal death directly. LRRK2 R1628P mutation increased the binding affinity of LRRK2 with Cyclin-dependent kinase 5 (Cdk5). Interestingly, R1628P mutation turned its adjacent amino acid residue S1627 on LRRK2 protein to a novel phosphorylation site of Cdk5, which could be defined as a typical type II (+) phosphorylation-related single nucleotide polymorphism. Importantly, we showed that the phosphorylation of S1627 by Cdk5 could activate the LRRK2 kinase, and neurons ectopically expressing R1628P displayed a higher sensitivity to 1-methyl-4-phenylpyridinium, a bioactive metabolite of environmental toxin MPTP, in a Cdk5-dependent manner.

**Conclusion:**

Our data indicate that Parkinson-related LRRK2 mutation R1628P leads to Cdk5 phosphorylation of LRRK2 at S1627, which would upregulate the kinase activity of LRRK2 and consequently cause neuronal death.

## Introduction

Although Parkinson’s disease (PD) was investigated intensely for ages, the pathogenesis of PD still remains indistinct. With the rapid growth of recent studies, genetic factors play a more and more important role in the progression of PD[1]. Some genes increase the risk of PD, such as α-synuclein (SNCA), parkin (PARK2), PTEN-induced putative kinase 1 (PINK1), oncogene DJ-1 (DJ-1), leucine-rich repeat kinase 2 (LRRK2) and ATPase type 13A2 (ATP13A2) have emerged from previous investigations[2, 3]. LRRK2 is a large and complex protein containing several distinct domains, including a leucine-rich repeat (LRR) domain, a Roc domain followed by its associated COR domain, a kinase domain, and a C-terminal WD40 domain[4, 5]. LRRK2 R1628P (c.4883G>C; rs33949390), within the COR domain, was found as the critical genetic risk factor for PD especially among Han-Chinese’s population in many previous studies[6–9]. However, the definite molecular mechanism about how R1628P variant lead to PD was still unclear.

The bioinformatics predictions using GPS 3.0, SCANSITE 3.0 and PhosSNP 1.0 suggested that R1628P mutation could turn its adjacent amino acid residues, serine 1627 (S1627) to a new candidate for phosphorylation by cyclin-dependent kinase 5 (Cdk5), one of the key kinases in the brain which was implicated to be dysregulated in several neurodegenerative diseases, including PD [10, 11] (S1 Fig). Our research focused on whether LRRK2 R1628P mutation altered the LRRK2 kinase activity, and caused subsequent neuronal death directly, or the pathogenic mechanisms of R1628P in PD involve a genotype-environment interaction, that R1628P genetic mutation of LRRK2 provided a potential two-hit target of environment toxic-induced Cdk5 activation.

## Results

### R1628P mutation do not alter the LRRK2 activity and cause neuronal toxicity directly

**Fig 1:**
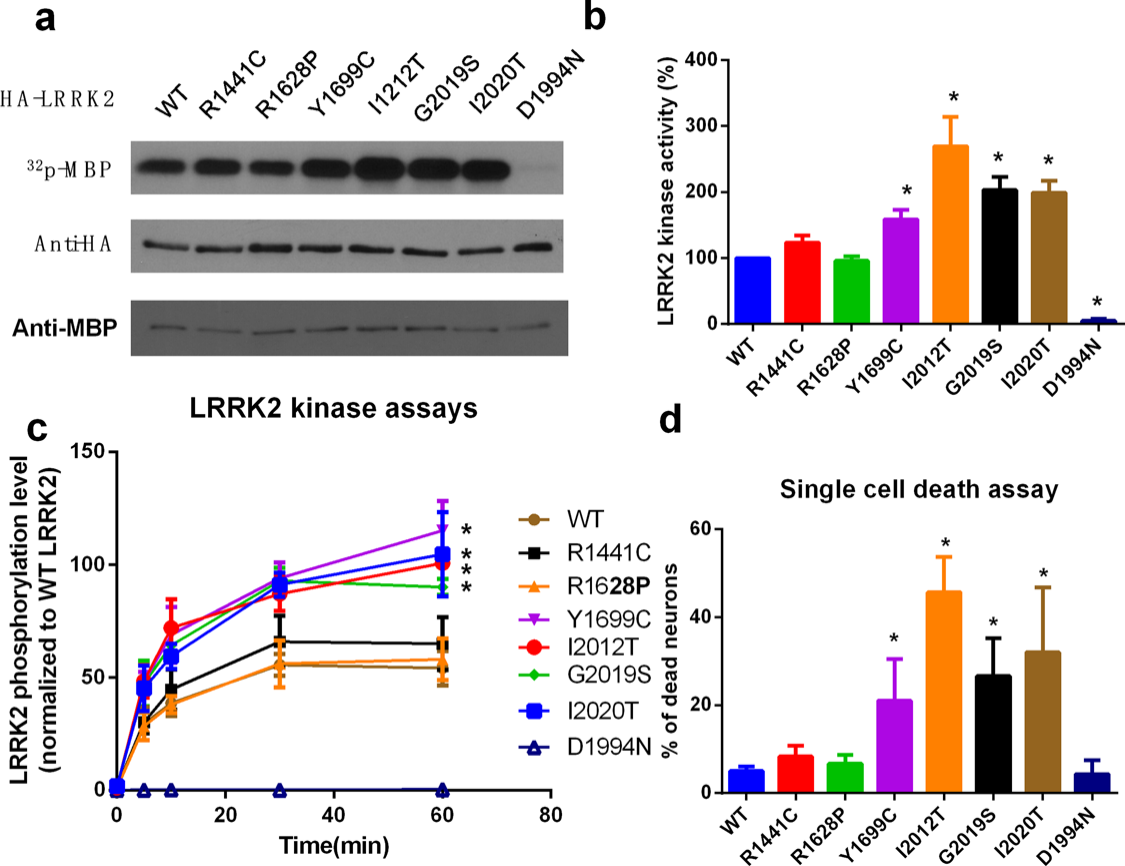
R1628P mutation do not alter the LRRK2 activity and cause neuronal toxicity directly. **a.** Kinase activity of LRRK2 mutants tested by kinase assay using MBP as substrate. HEK293 cells were transfected with HA-tagged WT LRRK2 or mutants in the Roc-COR-Kinase domain, including R1441C, R1628P, Y1669C, I2012T, G2019S, I2020T, and kinase-inactive mutant D1994N as indicated. After 24 h, LRRK2 was immunoprecipitated in the lysates by anti-HA antibody, and *in vitro* LRRK2 kinase assay was performed to measure the LRRK2 kinas activity using purified MBP protein as substrate. The bottom panel shows equal expression of HA-LRRK2 and MBP used in the lysates. **b.** The graph is the quantification of LRRK2 kinase activities in Fig 1A. The numbers are relative values, with WT set to 1. **c.** Kinase activity of LRRK2 mutants tested by kinase assay using LRRKtide as substrate. HA-tagged WT LRRK2 or mutants were immunoprecipitated as above, then *in vitro* LRRK2 kinase assay was performed using LRRKtide as substrate. **d.** Overexpression of R1628P mutation do not cause neuronal cell death. Primary-cultured cortical neurons were transfected with GFP-tagged WT LRRK2 or mutants in the Roc-COR-Kinase domain, including R1441C, R1628P, Y1669C, I2012T, G2019S, I2020T, and kinase-inactive mutant D1994N. After 48 h transfection, the dead cells were labeled with EthD-1 in red, and 200 GFP-positive neurons were counted to calculate the percentage of cell death. All the above results represent at least three independent experiments as the mean ± SD, *P<0.05, **P<0.01 (ANOVA).

To assess whether R1628P mutation alters the LRRK2 activity directly, we overexpressed wild-type (WT) and most studied LRRK2 mutants in the Roc-COR-Kinase domain (S2 Fig), including R1441C, R1628P, Y1669C, I2012T, G2019S, I2020T, and kinase-inactive mutant D1994N into HEK293 cells, the LRRK2 kinase activities were measured by *in vitro* kinase assay using MBP (Fig 1A and 1B) or LRRKtide (Fig 1C) as substrate, the results indicated that, unlike other mutants in the Roc-COR-Kinase domain, R1628P do not increase the LRRK2 kinase activity directly. Furthermore, we tested whether overexpression of wild-type or mutant LRRK2 could cause neuronal cell death. Primary-cultured cortical neurons were transfected with GFP-tagged WT or above mutants in the Roc-COR-Kinase domain and kinase-inactive mutant D1994N. After 48 h transfection, the dead cells were labeled with EthD-1 and counted to calculate the percentage of cell death. The results of the single cell death assay proved that, unlike other mutants in the Roc-COR-Kinase domain, introduction of R1628P mutation do not cause neuronal cell death directly (Fig 1D).

### Cdk5 could phosphorylate the adjacent S1627 in the LRRK2 R1628P mutant

**Fig 2:**
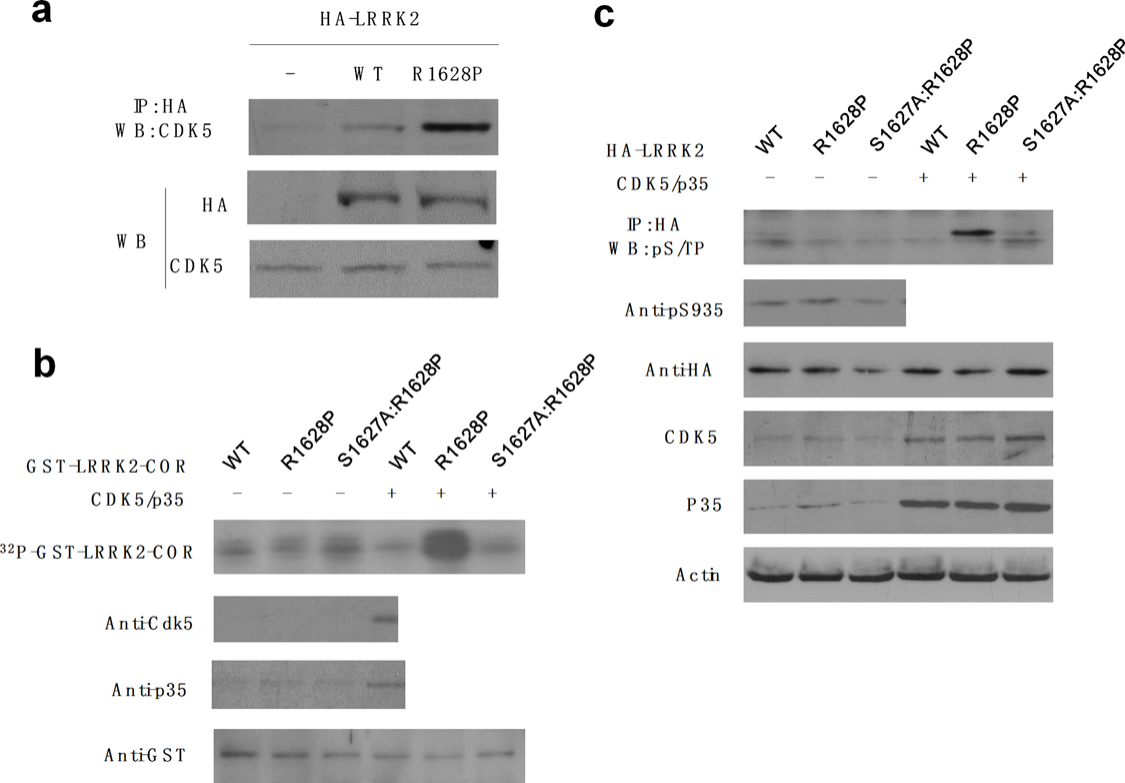
Cdk5 could phosphorylate the adjacent S1627 in the LRRK2 R1628P mutant. **a.** The R1628P mutation increase the binding affinity of LRRK2 with Cdk5. Primary-cultured cortical neurons were transfected with vehicle or LRRK2 (WT, R1628P). After 24 h, the exogenous LRRK2 was immunoprecipitated using anti-HA antibody, and the level of bound Cdk5 was measured by Western blotting. The bottom panel shows the loading control of HA-LRRK2 and Cdk5 used in the lysates. **b.** Cdk5 phosphorylates Serine 1627 (S1627) in R1628P mutant. Recombinant GST-tagged LRRK2 (COR domain, amino acids 1535~1878), including wild-type (WT), R1628P mutant and S1627A:R1628P double mutant, were purified. The GST-tagged LRRK2 recombinant protein were phosphorylated by active Cdk5/p35 and γ-^32^P-ATP *in vitro*, and the phosphorylation signals were analyzed by autoradiography. (top panel, about 60KD). The same membrane was probed with anti‑GST, Cdk5 or p35 antibody as a loading control (bottom panel). **c.** Cdk5 phosphorylates S1627 in cells. The HA-tagged LRRK2 (WT, R1628P and S1627A:R1628P) plasmids were cotransfected with Cdk5 and p35 in HEK293 cells. After 24 h of transfection, the LRRK2 were immuneprecipitated using an anti-HA antibody from lysates, and phosphorylation of LRRK2 were measured by Western blotting using a phospho-(Serine/Threonine)-Proline (pS/TP) antibody and anti-LRRK2 phospho S935 antibody. HA-LRRK2, Cdk5, p35, and actin levels were determined by Western blotting as a loading control.

Firstly, we confirmed the endogenous binding affinity of Cdk5 with LRRK2 in neurons (S3 Fig). Then, to measure the difference between wild-type or LRRK2 R1628P mutant, the primary cortical neurons were transfected with vehicle or LRRK2 (WT, R1628P), after 24 h of transfection, the exogenous LRRK2 was immunoprecipitated, and the level of bound Cdk5 was measured. The result showed that compared to the wild-type control, the R1628P mutation increase the binding affinity of LRRK2 with Cdk5 (Fig 2A). Secondly, we tested whether Cdk5 could phosphorylate S1627 in the R1628 mutant. We induced and purified the recombinant GST-LRRK2-COR (amino acids 1535~1878), including wild-type (WT), R1628P mutant and S1627A (mutation from Serine to Alanine to abolish the possibility of phosphorylation):R1628P double mutant, then a Cdk5 kinase assay was performed by incubating the GST-LRRK2 recombinant protein with active Cdk5/p35 and γ-^32^P-ATP, and the phosphorylation signals were analyzed by autoradiography. The results showed that only R1628P mutant could be phosphorylated by Cdk5, but not the WT and S1627A:R1628P double mutant (Fig 2B). We also repeated and confirmed the above findings in cells. The HA-tagged LRRK2 (WT, R1628P and S1627A:R1628P) plasmids were cotransfected with Cdk5 and p35 into HEK293 cells, after 24 h of transfection, the LRRK2 were immunoprecipitated from lysates, and phosphorylation of LRRK2 were measured by Western blotting using a phospho-(Serine/Threonine)-Proline (pS/TP) antibody. The data showed the similar result with the *in vitro* experiment, which indicated that Cdk5 could phosphorylate S1627 in a R1628P mutation dependent manner (Fig 2C), while dominant negative form of Cdk5 couldn’t phosphorylate S1627 in a R1628P mutant (S4 Fig.). To test the specificity of Cdk5 phosphorylation of LRRK2 R1628P on S1627, we tested the S935 phosphorylation, which is phosphorylated by PKA [12], the resulted showed the S935 isn’t phosphorylated by Cdk5/p35 (Fig 2C).

### Phosphorylation of R1628P mutant by Cdk5 increases the LRRK2 kinase activity and causes cell death

**Fig 3:**
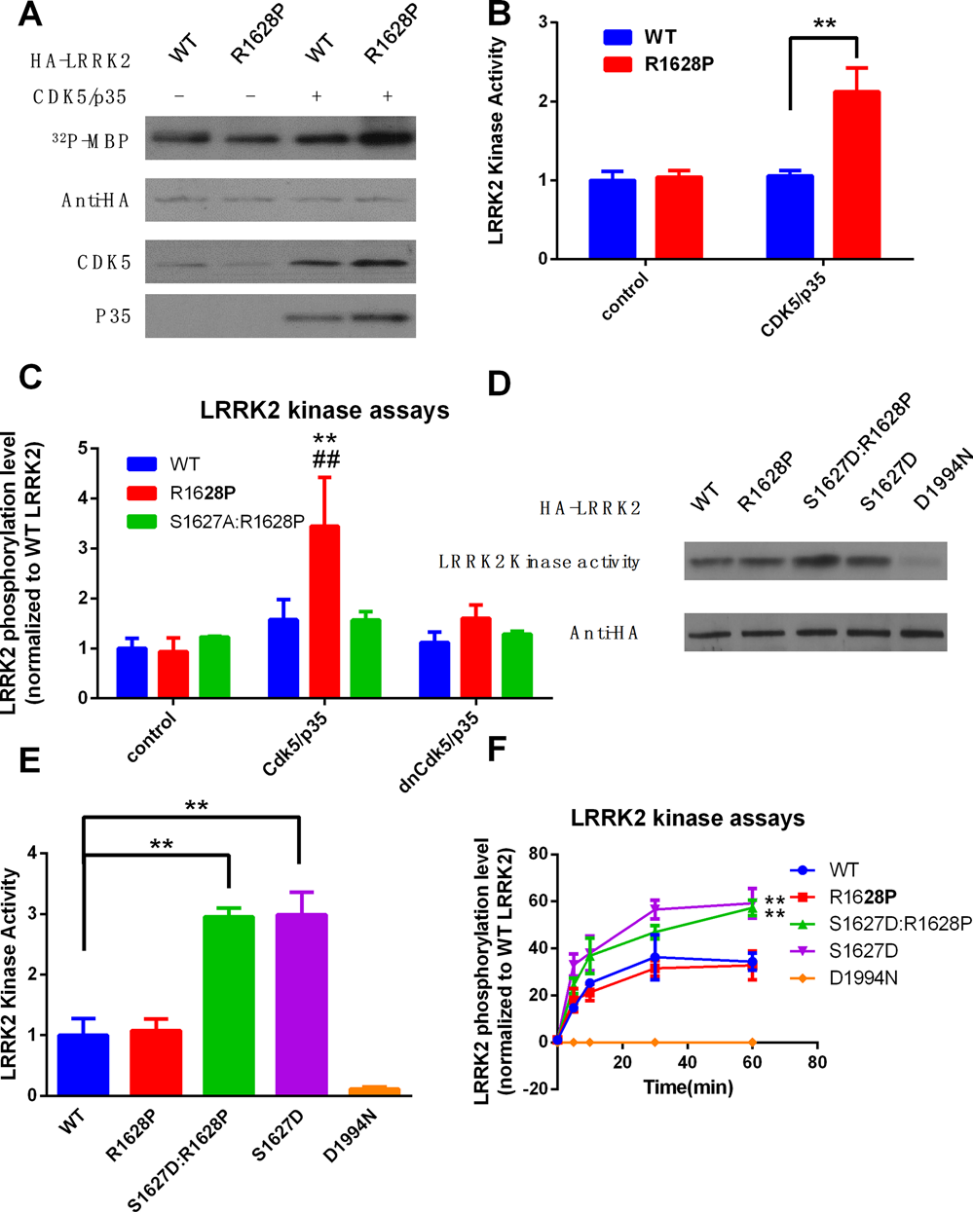
Phosphorylation of R1628P mutant by Cdk5 increases the LRRK2 activity and causes cell death. **a.** Plasmids of HA-tagged wild-type (WT) or R1628P mutant LRRK2 were cotransfected with Cdk5/p35 in HEK293 cells, as indicated. After 24 h, LRRK2 was immune precipitated in the lysates by anti-HA antibody, and *in vitro* LRRK2 kinase assay was performed to measure the LRRK2 kinas activity using purified MBP protein as substrate. HA-LRRK2, Cdk5, and p35 levels were determined by Western blotting as a loading control. **b.** The graph is the quantification of LRRK2 kinase activities in Fig 3A. The numbers are relative values, with WT w/o Cdk5/p35 set to 1. **c.** Plasmids of HA-tagged wild-type (WT), R1628P, and S1627A:R1628P mutant LRRK2 were cotransfected with Cdk5 or dominant-negative form of Cdk5 (dnCdk5) and p35 in HEK293 cells, as indicated. After 24 h, LRRK2 was immune precipitated in the lysates by anti-HA antibody, and *in vitro* LRRK2 kinase activity was measured for 30min using LRRKtide as substrate. The results represent as the mean ± SD, **P<0.01 (compared with WT:control group) ##P<0.01 (compared with WT:Cdk5/p35 group) (ANOVA). **d.** Phosphorylation mimic of S1627 (S1627D) increased the LRRK2 kinase activity. HEK293 cells were transfected with HA-tagged LRRK2 plasmids, including wild-type (WT), R1628P, S1627D and S1627D:R1628P double mutant. LRRK2 kinase activities were measured as above. **e.** The graph is the quantification of LRRK2 kinase activities in Fig 3D. The numbers are relative values, with WT set to 1. **P<0.01 (ANOVA) **f.** HEK293 cells were transfected with HA-tagged LRRK2 plasmids, including wild-type (WT), R1628P, S1627D and S1627D:R1628P. LRRK2 kinase activities were measured as above for indicated period of time using LRRKtide as substrate. **P<0.01 (compared with WT group, ANOVA)

Furthermore, we explored the molecular and cellular function of Cdk5 phosphorylation of S1627 in the R1628P mutant. Plasmids of WT or R1628P LRRK2 were cotransfected with Cdk5/p35 in HEK293 cells, as indicated. After 24 h, LRRK2 was immunoprecipitated in the lysates by anti-HA antibody, and *in vitro* LRRK2 kinase assay was performed using MBP or LRRKtide as substrate. The results showed that Cdk5 cotransfection could increase the kinase activity of R1628P mutant but not WT LRRK2 or S1627A:R1628P double mutant (Fig 3A, 3B and 3C). To assess whether activation of LRRK2 by phosphorylation on R1627 was dependent on R1628P mutation, we transfected LRRK2 plasmids, including WT, R1628P, S1627D (mutation from Serine to Aspartic acid to mimic the phosphorylation of serine), S1627:R1628P double mutant, and kinase inactive form LRRK2 D1994N into HEK293 cells, then LRRK2 kinase activities were measured as above. The results showed that the LRRK2 kinase activity could be increased by the phosphorylation mimic of S1627 (S1627D), even without R1628P mutation (Fig 3E, 3F and 3G).

### Neurons ectopically expressing R1628P displayed a higher sensitivity to MPP+ in a Cdk5-dependent manner

**Fig 4:**
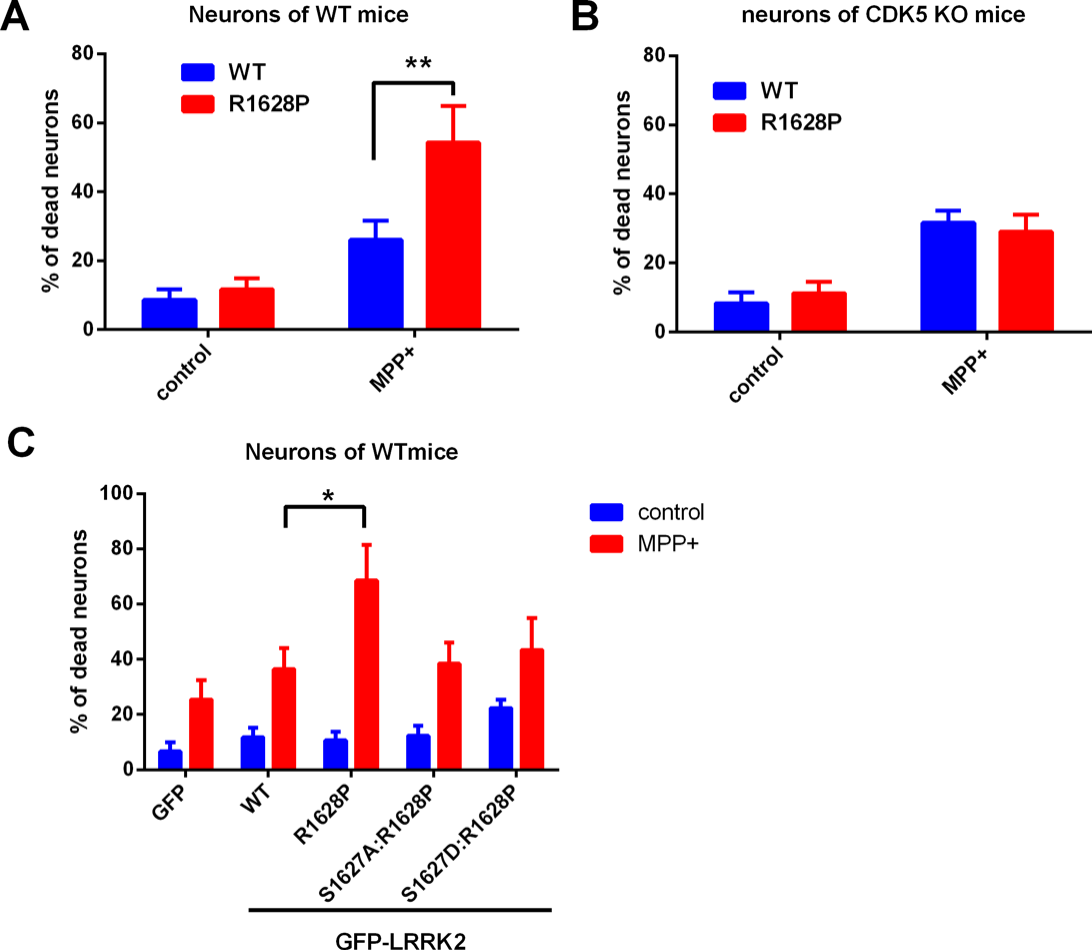
Neurons ectopically expressing R1628P displayed a higher sensitivity to MPP+ in a Cdk5-dependent manner. **a.** Neurons with the R1628P mutation display a higher sensitivity to MPP+. Primary-cultured cortical neurons from wild-type mice were transfected with GFP-tagged wild-type (WT) or R1628P mutant LRRK2 plasmids for 24 h, and then exposed to MPP+ (30 μM) for 24 h. The dead cells were labeled with EthD-1 in red, and 200 GFP-positive neurons were counted to calculate the percentage of cell death. **b.** Cdk5 deletion protects the neurons with the R1628P mutation from MPP+ toxicity. The above procedures were performed in primary-cultured cortical neurons from the neuronal Cdk5 conditional knockout mice. Single cell survival assay was conducted as above. The results represent at least three independent experiments as the mean ± SD, **P<0.01 (ANOVA). **c.** The higher sensitivity of R1628P to MPP+ requires the phosphorylation of S1627 on LRRK2. Primary-cultured cortical neurons from wild-type mice were transfected with GFP vector, GFP-tagged wild-type (WT) LRRK2 or R1628P, S1627A:R1628P, S1627D:R1628P mutant for 24 h, and then exposed to MPP+ (30 μM) for 24 h. The dead cells were labeled with EthD-1 in red, and 200 GFP-positive neurons were counted to calculate the percentage of cell death. The results represent at least three independent experiments as the mean ± SD, *P<0.05, (ANOVA)

Finally, we observed the effect of R1628P mutation on neuronal cell death in PD cell model. Neurons from WT or Cdk5 conditional knockout mice (S5 Fig) were transfected with GFP-tagged WT or R1628P LRRK2 plasmids, and then exposed to MPP+, a bioactive metabolite of environmental neurotoxin MPTP. The dead cells were labeled and GFP-positive cells was counted to calculate the percentage of cell death. The neurons with the R1628P mutant display a higher sensitivity to MPP+, while Cdk5 deletion protects the neurons with the R1628P mutant from MPP+ toxicity (Fig 4A and 4B). We tested the toxicity of LRRK2 (WT, R1628P, S1627A:R1628P, S1627D:R1628P) with or without MPP+ treatment in primary-cultured neurons from wild-type mice. The results proved that the higher sensitivity of R1628P mutant to MPP+ is through the phosphorylation of S1627 by Cdk5 (Fig 4C).

## Discussion

Elucidation of the effect of the familial mutations in LRRK2 on its functions or kinase activities is crucial to the understanding of how these mutations cause neurodegeneration in PD. LRRK2 may play a role in the phosphorylation of proteins central to PD, the ezrin/radixin/moesin (ERM) family proteins[13], α-synuclein[14], microtubule-associated protein Tau[15], and Endophilin A[16] have been characterized to be phosphorylated by LRRK2. Kinase substrates of LRRK2 kinase assay have still not been independently confirmed, while MBP, LRRKtide or autophosphorylation of LRRK2(1491, 1503, 2483, etc) were mostly used substrates in LRRK2 kinase assays [17]. Although a clear picture has yet to emerge, data from a number of groups has highlighted that the upregulation of the kinase activity of LRRK2 is the key pathogenic phenotype of mutations linked to PD. Some mutant forms of LRRK2 in the catalytic core have been proved to increase the kinase activity and mediate the consequent neuronal toxicity directly. The tandem Roc-COR-Kinase domain arrangement suggests that their activities might be coupled, and the GTPase activity of Roc might modulate the kinase activity. Several studies have shown that GTP binding to the Roc domain regulates the kinase activity of LRRK2, and R1441C/G/H (mutations in the Roc domain) has been shown to up-regulate kinase activity [18, 19]. The molecular pathology of mutations in COR domain is rarely discussed. The Y1699C substitution in the COR domain was proved to disrupt LRRK2 interactions with DVL1, DVL2 and DVL3 and increase kinase activity [20, 21] another R1728H/L substitutions have not been proved to be pathogenic[22]. In previous studies, only G2019S mutation of LRRK2 shows consistently an increase in LRRK2 activity, and all other investigated LRRK2 mutations show variable results on kinase activity and toxicity by different investigators [23].

But until now, little is known about function of R1628P and the influence of the R1628P mutation on LRRK2 kinase activity. Herein, we found that R1628P substitution did not alter the LRRK2 kinase activity, and cause neuronal death directly. While, R1628P mutation turned its adjacent amino acid residue S1627 to a new phosphorylation site of Cdk5. Moreover, Cdk5 phosphorylation of S1627 in the R1628P mutant increases the LRRK2 kinase activity. We also proved that neurons ectopically expressing R1628P displayed a higher sensitivity to MPP+, the bioactive metabolite of environmental neurotoxin MPTP, and Cdk5 deletion by conditional knockout technique protects the neurons with R1628P mutation from MPP+ toxicity. More and More epidemiological studies have suggested a possible link between environmental toxin (such as MPTP, rotenone, and paraquat) exposure and PD [24, 25]. Recent evidences point towards a putative role of mitochondrial dysfunction and oxidative stress in PD [26, 27]. In the current study, we indicated that a mechanism of gene-environment interaction and “two-hot” model were involved in the progress of PD patients with genetic R1628P mutation, that the R1628P mutation do not alter the LRRK2 kinase activity and cause neuronal death per se, but rather increases the susceptibility of carriers to environment toxins or aging stress, which consequently activated Cdk5[10, 11].

It’s worth mentioning that *LRRK2* c.4883G>C (R1628P) variant is as a typical phosphorylation-related SNP (phosSNP). A phosSNP is a non-synonymous SNPs that might influence protein phosphorylation status, which was defined using genome-wide bioinformatics analysis in 2009[25, 26]. PhosSNPs can be classified into five groups: 1) Type I: directly adding (+) or removing (-) phosphorylation sites; 2) Type II: creating (+) or disrupting (-) adjacent phosphorylation sites; 3) Type III: changing PK types for adjacent phosphorylation sites; 4) Type IV: occurring at phosphorylation sites to induce changes of PK Types; 5) Type V: removing following phosphorylation sites by nonsense SNPs[28, 29]. Ren et al. reported that ~70% of non-synonymous SNPs in human genome are potential phosSNPs [28]. They reported a total of 64,035 phosSNPs, among which 2,004 are experimentally validated. For example, the K897T mutation on human ERG1/KCNH2/Kv11.1 protein creates a new AKT phosphorylation site to prolong the QT interval of cardiac myocytes [30]. Li et al. observed that the P47S mutation of p53 strongly compromises the phosphorylation level of its adjacent residue Serine 46 by p38 MAPK and reduces the ability of p53 to induce apoptosis[31]. Moreover, the D149G mutation on p21WAF1/CIP1 could attenuate Serine 146 phosphorylation by PKCδ to resist tumor necrosis factor α-induced apoptosis and play an important role in cancer development[32]. In our case, the R1628P substitution turns its adjacent Serine 1627 to a new phosphorylation site of Cdk5, that defines *LRRK2* c.4883G>C (R1628P) variant as a Type II (+) phosSNP.

**Fig 5:**
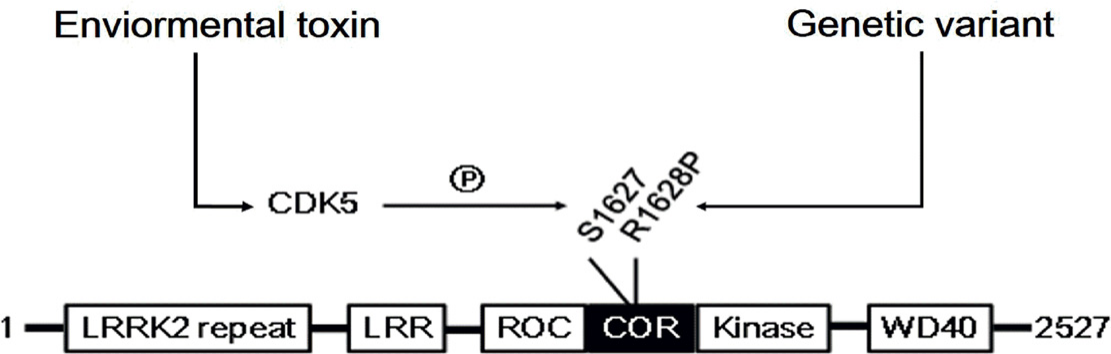
The Schematic diagram shows that the R1628P genetic mutation of LRRK2 provide a potential “two-hit” target of environment toxic MPP+-induced Cdk5 activation, and Cdk5 could phosphorylate the adjacent amino residue S1627 of R1628P mutation, thus activate LRRK2 kinase activity and cause neuronal death.

Together, in the current study, we identified that, in the R1628P mutant, its adjacent amino residue S1627 could be a potential phosphorylation site of Cdk5. Rather than upregulating the kinase activity and neuronal toxicity directly, the R1628P genetic mutation of LRRK2 provide a potential “two-hit” target of environment toxic-induced Cdk5 activation (Fig 5). While, the relevance for the findings in intact brain and for Parkinson’s disease in patients requires further evaluation. Our findings elucidate the role of genotype-environment interaction-related LRRK2 R1628P mutation in neuronal death in LRRK2-linked PD in Han-Chinese population and provide a novel therapeutic target for drug design or genetic modulation.

## Materials and Methods

### Chemicals and antibodies

For immunoblotting, anti-Phosphothreonine-Proline/Phosphoserine-Proline (pS/TP) were purchased from Abcam; rabbit polyclonal anti-actin, mouse monoclonal anti-GST, rabbit polyclonal anti-HA were all from Cell Signaling; polyclonal anti‑p35 (N‑20) from Santa Cruz; mouse monoclonal anti-Cdk5 from Neomakers, anti-phospho S935 LRRK2 and total LRRK2 antibody are from ThermoFisher. Antibodies were used at dilutions recommended by the manufacturers. 1-methyl-4-phenylpyridinium (MPP+), adenosine-triphosphate (ATP) were obtained from Sigma-Aldrich. ATP Gamma 32P (γ-^32^P-ATP) was from Perkin Elmer. Active Cdk5/p35 was purchased from Upstate Biotechnology. Purified MBP protein was from EMD Millipore. LRRKtide (RLGRDKYKTLRQIRQ) was synthesized by Shenggong Corp. (Shanghai, China).

### Constructs and recombinant proteins

Constructs encoding full-length (FL) HA-tagged LRRK2, including R1441C, Y1669C, I2012T, G2019S, I2020T, and kinase-inactive mutant D1994N were obtained from Addgene (Deposited by Michael J Fox Foundation). HA-tagged and GFP-tagged LRRK2 with R1628P, S1627A:R1628P, S1627D, and S1627D:R1628P mutations were performed using QuikChange site-directed II mutagenesis kit (Stratagene), and the point mutations were confirmed by sequencing. GST-tagged LRRK2-COR (COR domain, amino acids 1535~1878) was obtained by PCR using FL-HA-LRRK2-WT as template and subcloned to pGEX-2T1 vector (Addgene). Recombinant GST-tagged LRRK2-COR protein were induced and purified from BL21 (DE3) competent cells (Invitrogen).

### Animals

Cdk5 conditional knockout (ko) mice in the forebrain were generated by the crossbreed between B6.129S4(Cg)-Cdk5tm1.1Lht/J (Cdk5-loxP) and CamKIIα-Cre mice. Cdk5-loxP mice were purchased from Jackson Laboratories. CamKIIα-Cre mice was a gift from Southern Medical University. All knockout mice were routinely genotyped by standard PCR according to the genotyping protocols database of The Jackson Laboratory website. All animal experiments were performed in compliance with the institutional guidelines for the care and use of animal research. The protocol was approved by the Animal Care and Use Committee of Tongji Medical College. The mice were group housed in the Animal Core Facility of Tongji Medical College under specific pathogen-free conditions at a controlled temperature of 23±2°C, relative humidity 55±10%. The mice were given food and water ad libitumwith a 12-hr light dark cycle. Mice were normally euthanized by CO2 inhalation at the end of a study.

### Cell culture and plasmid transfection

HEK293 cells were purchased from China Center for Type Culture Collection, CCTCC, and cultured in DMEM supplemented with 10% FBS. Primary cortical neurons were cultured from mice at embryonic day 16–18 (E16-18) on six-well plates coated with poly-L-lysine. Neurons were maintained in Neurobasal medium (Invitrogen) containing 2% B27 supplement, 0.5 mM glutamine and 25 mM glutamate. MPP+ treatments or plasmid transfection were performed 7 days after plating. Primary cortical neurons or HEK293 cells were transfected using Lipofectamine 2000 reagents (Invitrogen).

### Immunoprecipitation and immunoblotting

Lysates were generally prepared with NP‑40 lysis buffer (20 mM Tris, pH 8.0, 135 mM NaCl, 1 mM MgCl2, 0.1 mM CaCl2, 10% glycerol, 1% NP‑40, 0.1 mM Na3VO4, 50 mM β‑glycerol phosphate, 10 mM NaF and protease inhibitors from Roche). For immunoblotting by SDS/PAGE analysis, 50–100 μg of protein was used and 100–500 μg of protein was used for immunoprecipitation, incubated with 1–2 μg of the corresponding antibody at 4°C overnight. The immunocomplexes were collected with protein G plus/protein A‑agarose (Calbiochem) and washed three times with NP‑40 lysis buffer.

### *In vitro* LRRK2 and Cdk5 kinase assay

LRRK2 kinase activity was measured by *in vitro* LRRK2 kinase assay using MBP or LRRKtide as substrate [17]. For the kinase assays using MBP as substrate, LRRK2 was immunoprecipitated from cell lysates, and the corresponding immunoprecipitates were washed twice with LRRK2 kinase buffer (50 mM HEPES, pH 7.5, 150 mM NaCl, 4 mM MnCl_2_, 10% glycerol, 1 mM dithiothreitol, and 100 μM Na_3_VO_4_) and incubated in the kinase buffer containing γ‑^32^P-ATP and 1 μg of purified MBP for 30 min at 30°C. The reaction was stopped with SDS sample buffer and boiling for 5 min. Phosphorylation of MBP was analyzed by SDS–PAGE and autoradiography. For the LRRKtide peptide kinase assays, LRRK2 was immunoprecipitated from the cell lysate, then in vitro kinase assays were performed as above, in triplicate, using LRRKtide peptides as substrate. LRRKtide peptide was incubated at final concentration 10 μM in the kinase buffer containing γ‑^32^P-ATP for 60 min at 30°C. Reactions were stopped by spotting on P81 phosphocellulose paper (Whatman), washed five times for 5 min each in 75 mM phosphoric acid and then washed once for 5 min in acetone. Air-dried papers were counted on a scintillation counter (Beckman Coulter LS 6500). Counts were converted to moles of total phosphate using a standard curve.

To test whether LRRK2 was phosphorylated by Cdk5, we performed *in vitro* Cdk5 kinase assay according to the manufacturer’s instructions. Active Cdk5/p35 kinase was purchased from Upstate Biotechnology. Briefly, 2 μg of purified GST-LRRK2-COR recombinant protein was incubated with active Cdk5/p35 kinase in Cdk kinase buffer (8 mM MOPS/NaOH pH7.0, 200 nM EDTA) containing 20 μM ATP, 10 μCi of γ‑^32^P-ATP for 15 min at 30°C. The reaction was stopped with SDS sample buffer and boiling for 5 min. Phosphorylation of MBP was analyzed by SDS–PAGE and autoradiography.

### Single Cell death assay

Single-cell survival assays were performed using a Viability/Cytotoxicity Kit (Invitrogen) according to the manufacturer’s instructions. Briefly, primary-cultured cortical neurons were transfected with GFP-tagged vehicle or LRRK2 plasmids for 48 h. For experiments with MPP+ treatment, the neurons were exposed to MPP+ (30 μM) for another 24 h after 24 h of transfection. Neurons were stained with ethidium homodimer‑1 (EthD‑1) without permeabilization. GFP-positive cells with or without EthD‑1 staining were counted using an Olympus IX51 fluorescence microscope in a blinded manner. Two hundred or more transfected cells were counted for each treatment. Numbers of dead CGNs were calculated as a percentage of cells with colocalization of EthD-1 and GFP in the total number of GFP-positive cells counted.

### Statistical analysis

All molecular and cellular results were analyzed by ANOVA. All data are presented as the mean ±SD. All results are representative of at least three independent experiments. A P value less than 0.05 was considered to be statistically significant. *P<0.05.

## Supporting Information

S1 Fig The bioinformatics predictions using GPS 3.0 software suggested that R1628P mutation could turn its adjacent amino acid residues S1627 to a new candidate for phosphorylation by Cdk5. **a.** the prediction result of Cdk5 phosphorylation candidate site on wild-type LRRK2; **b.** the prediction result of Cdk5 phosphorylation candidate site on LRRK2 R1628P mutant.
(JPG)

S2 Fig LRRK2 domain structure and the position of PD-linked point mutations. (JPG)

S3 Fig The endogenous binding affinity of Cdk5 with LRRK2 in neurons. The endogenous LRRK2 was immunoprecipitated from the cell lysates of primary cortical neurons using anti-LRRK2 antibody or irrelevant IgG as negative control, then the level of bound Cdk5 and immunoprecipatated LRRK2 was measured. The level of Cdk5 and LRRK2 was measured in the cell lysates of primary cortical neurons by Western blotting as a input control.
(JPG)

S4 Fig Cdk5, but not its kinase dead form dnCdk5, could phosphorylate S1627 in a R1628P mutation dependent manner. The HA-tagged LRRK2 (WT, R1628P) plasmids were cotransfected with Cdk5 or dominant-negative Cdk5 (dnCdk5) and p35 in HEK293 cells. After 24 h of transfection, the LRRK2 were immunoprecipitated using an anti-HA antibody from lysates, and phosphorylation of LRRK2 were measured by Western blotting using a phospho-(Serine/Threonine)-Proline (pS/TP) antibody and anti-LRRK2 phospho S935 antibody. HA-LRRK2, Cdk5, p35, and actin levels were determined by Western blotting as a loading control.
(JPG)

S5 Fig Protein levels of Cdk5, p35 and LRRK2 in primary-cultured cortical neurons from wild-type (WT) or Cdk5 conditional knockout (KO) mice were measured by Western blotting. (JPG)

## Abbreviations

PD: Parkinson’s disease
LRRK2: leucine-rich repeat kinase 2
LRR: leucine-rich repeat
MPP+: 1-methyl-4-phenylpyridinium
MPTP: 1-methyl-4-phenyl-1,2,3,6-tetrahydropyridine
ATP: adenosine-triphosphate
Cdk5: cyclin-dependent kinase 5
FL: full-length
KO: knockout
WT: wild type
EthD‑1: ethidium homodimer‑1
GFP: green fluorescent protein
