# Supplementary figures and images for: Parkinson-Related LRRK2 Mutation R1628P Enables Cdk5 Phosphorylation of LRRK2 and Upregulates Its Kinase Activity
Source: PLoS One. 2016 Mar 1;11(3):e0149739. doi: 10.1371/journal.pone.0149739 (PMC4773127; doi:10.1371/journal.pone.0149739)

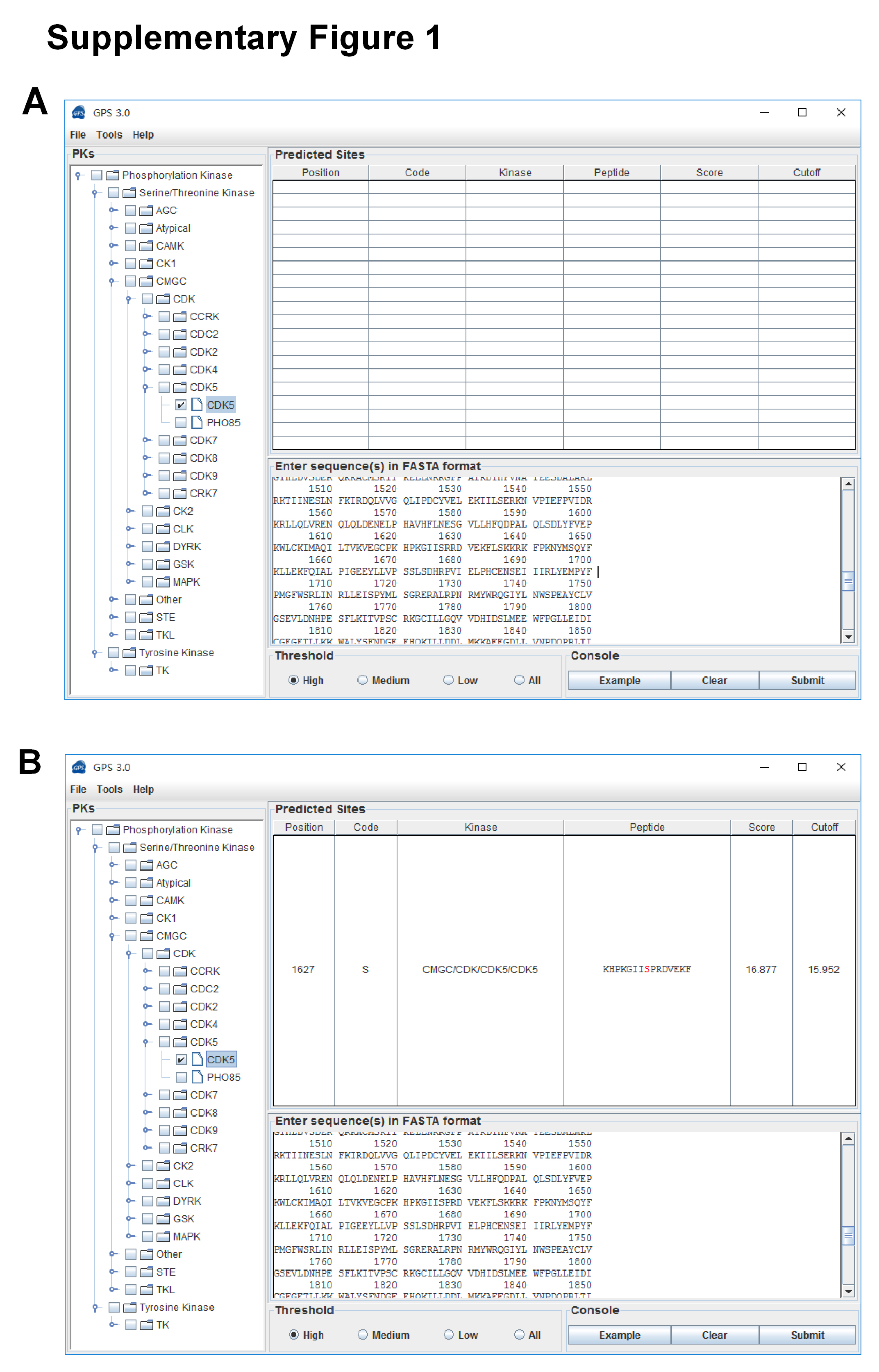

Supplement: S1 Fig — a. the prediction result of Cdk5 phosphorylation candidate site on wild-type LRRK2; b. the prediction result of Cdk5 phosphorylation candidate site on LRRK2 R1628P mutant. (JPG) [file pone.0149739.s001.jpg]

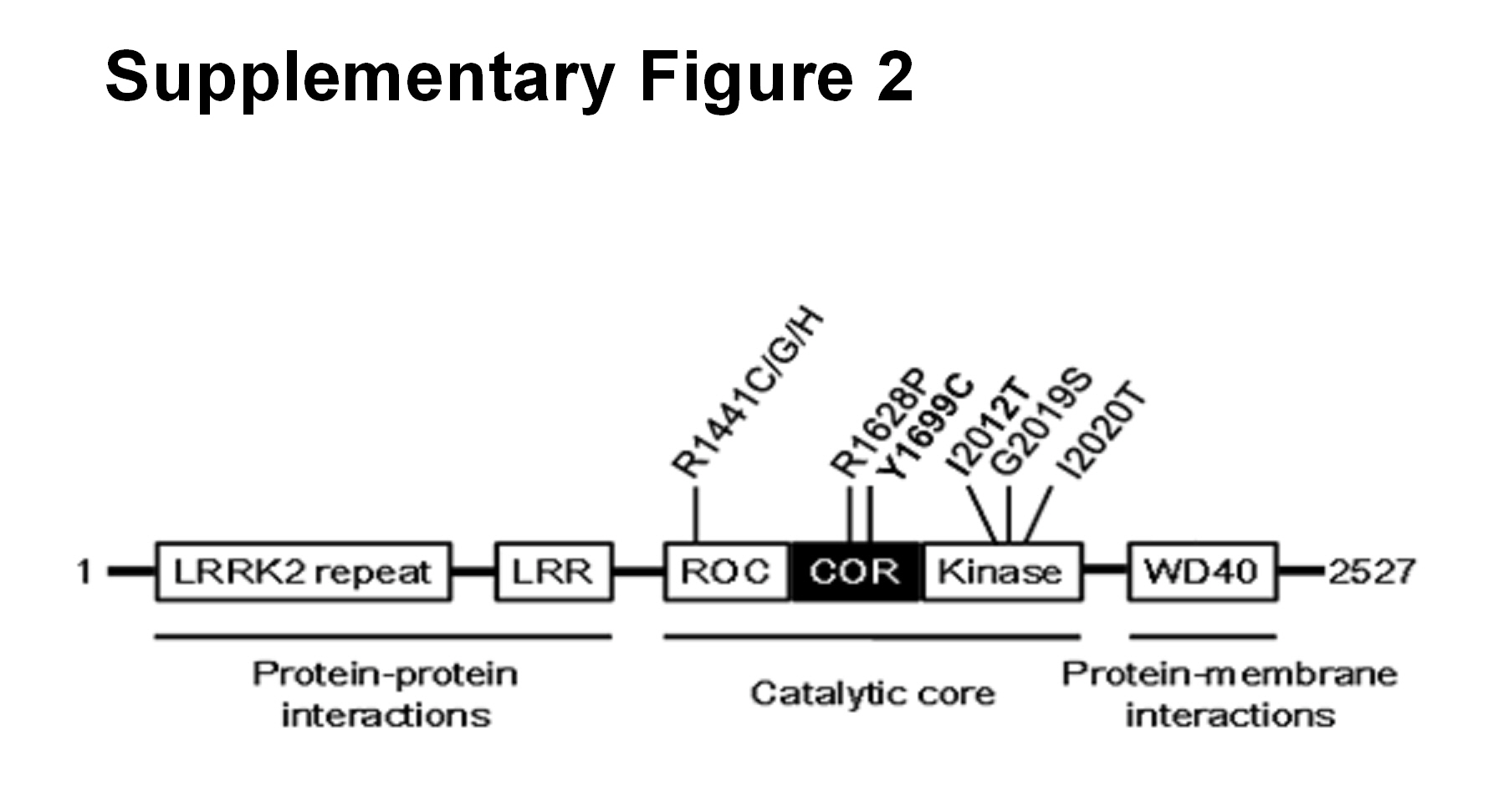

Supplement: S2 Fig — (JPG) [file pone.0149739.s002.jpg]

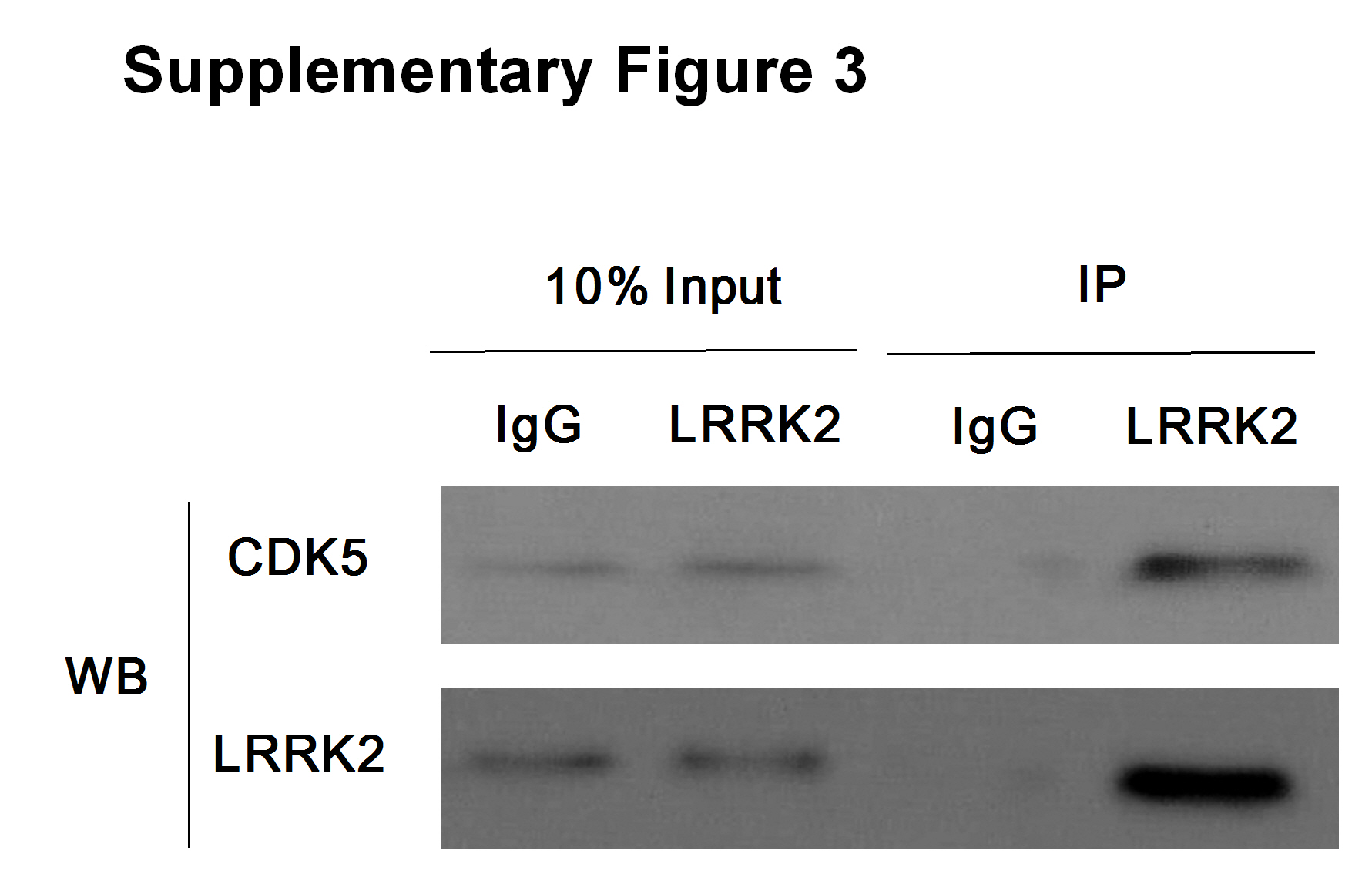

Supplement: S3 Fig — The endogenous LRRK2 was immunoprecipitated from the cell lysates of primary cortical neurons using anti-LRRK2 antibody or irrelevant IgG as negative control, then the level of bound Cdk5 and immunoprecipatated LRRK2 was measured. The level of Cdk5 and LRRK2 was measured in the cell lysates of primary cortical neurons by Western blotting as a input control. (JPG) [file pone.0149739.s003.jpg]

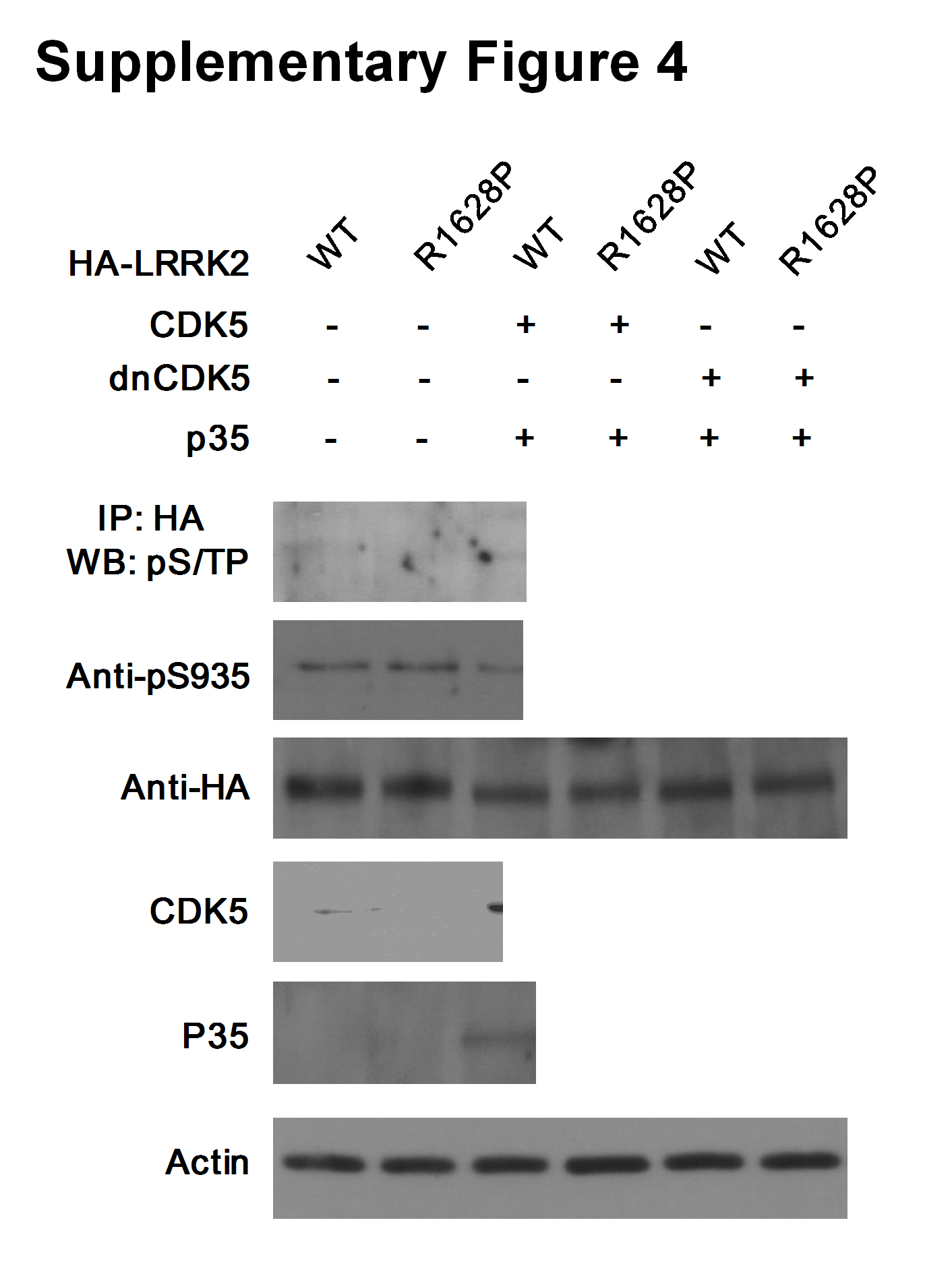

Supplement: S4 Fig — The HA-tagged LRRK2 (WT, R1628P) plasmids were cotransfected with Cdk5 or dominant-negative Cdk5 (dnCdk5) and p35 in HEK293 cells. After 24 h of transfection, the LRRK2 were immunoprecipitated using an anti-HA antibody from lysates, and phosphorylation of LRRK2 were measured by Western blotting using a phospho-(Serine/Threonine)-Proline (pS/TP) antibody and anti-LRRK2 phospho S935 antibody. HA-LRRK2, Cdk5, p35, and actin levels were determined by Western blotting as a loading control. (JPG) [file pone.0149739.s004.jpg]

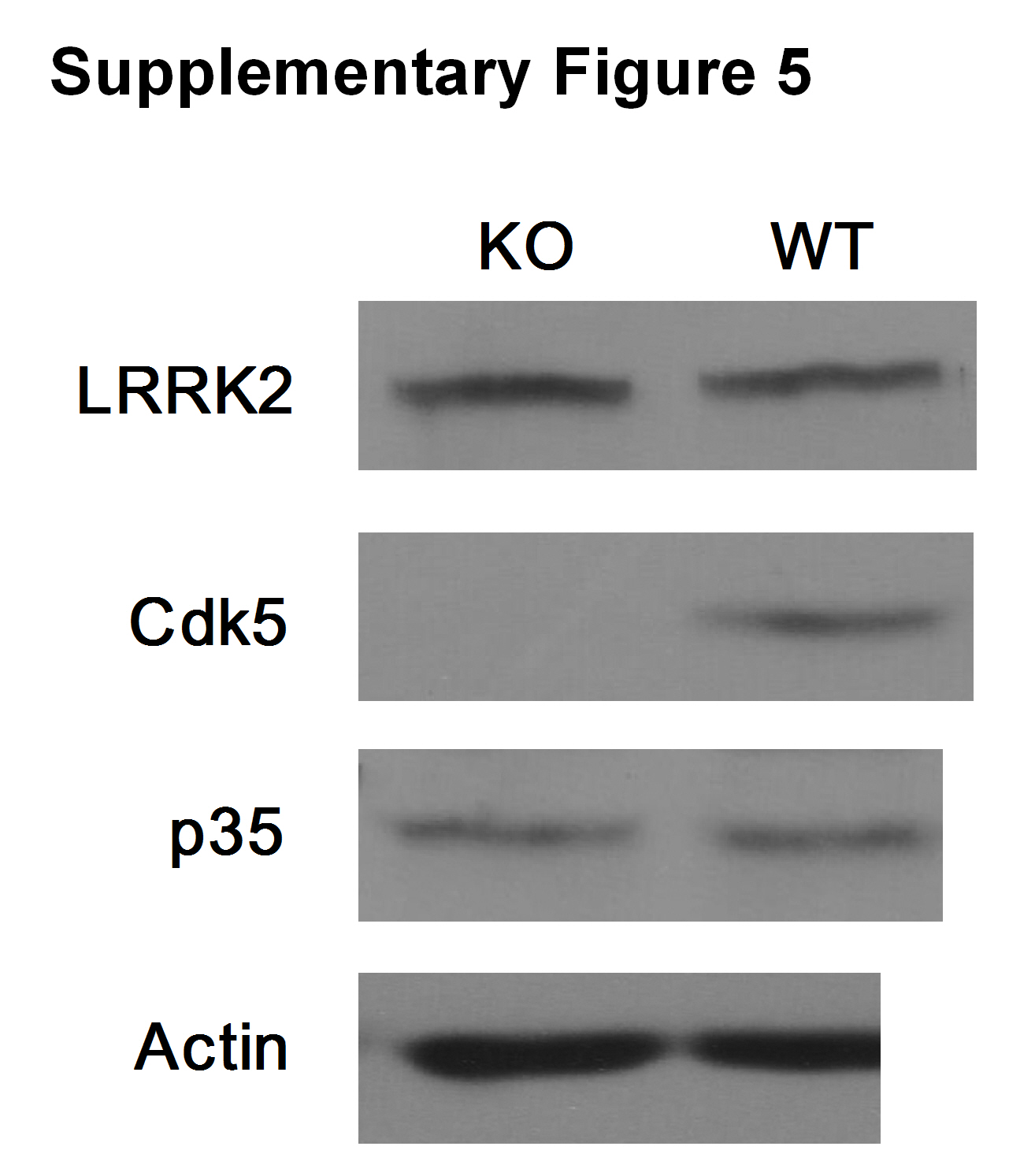

Supplement: S5 Fig — (JPG) [file pone.0149739.s005.jpg]
